# Contrast affects stimulus detection in natural scenes

**DOI:** 10.3389/fnhum.2025.1553504

**Published:** 2025-05-15

**Authors:** Gabriel Lopes, Mara Tavares, Catarina Mendonça

**Affiliations:** ^1^Department of Psychology, University of the Azores, Ponta Delgada, Portugal; ^2^University Research Center in Psychology, University of Algarve, Faro, Portugal

**Keywords:** contrast, detection, shape perception, shape discrimination, timing, position

## Abstract

How can we predict if a brief stimulus will be detected or perceived when embedded in a dynamic natural scene, such as those we encounter in our daily lives? This is a complex problem, with several approaches to it. Here, we were interested in determining the minimum luminance contrast to the background scene required to achieve detection and shape perception. To investigate this, we used natural videos with briefly appearing natural events, varying in timing of appearance, shape, position, and contrast. We found that there is an interplay between the timing of the event, its position, and the contrast needed for detection. However, for correct shape perception, timing was not a relevant variable. A lower contrast was required for event detection than for correct shape perception. We conclude that contrast alone can affect stimulus detection, but other parameters might interact with it in this task.

## Introduction

The problem of predicting if a stimulus will be detected in a natural environment is complex. In everyday life, we encounter a wide variety of visual objects, and the ability to detect and distinguish between them is crucial to our efficiency. For example, when driving on an unlit road at night, a pedestrian without a reflective vest might appear. It is crucial to detect them as quickly as possible. Several factors might play a role in our ability to detect and understand a stimulus in a natural scene, such as size, duration, luminosity and contrast (Green, 2020).

Contrast refers to the difference in brightness between a given object and the surrounding background (Harley et al., 2004). Several studies have explored how varying levels of contrast affect our ability to detect and identify stimuli. Pelli and Bex (2013) highlights the fundamental role of contrast in object and stimulus detection, reinforcing its importance as a key factor in visual perception. However, while their study establishes a strong foundation for understanding contrast sensitivity, it primarily focuses on controlled experimental conditions and does not explore contrast detection in dynamic, real-world environments.

Contrast and color affect target detection, with brighter stimuli with higher color contrast becoming more detectable compared to the background (Park et al., 2017). When studying the detection of low-contrast patterns at different luminance settings, Sund et al. (2008) found that, at luminosities between 1 and 350 cd/m^2^, twice the contrast is required to detect a dark object on a gray background compared to a dark object on a dark background. They also found that ambient light has some moderate effects on contrast detection thresholds.

The detection threshold is the point at which the stimulus begins to be detected by the sensory system, i.e., the lowest intensity of a stimulus that the individual can perceive. This detection threshold is subjective and can vary depending on several factors, such as luminance, stimulus size, subject age and stimulus duration (Green, 2020). In addition to these factors, attentional asymmetries can also influence stimulus detection. Hartmann et al. (2019) investigated the phenomenon of pseudoneglect, where individuals tend to initially explore the left side of the space. Their study found that this leftward bias occurs regardless of the distance of the stimulus but dynamically redistributes over time. Similarly, Iyilikci et al. (2010) found a left-side advantage in change detection tasks. These findings suggest that stimulus positioning within a scene may systematically affect its likelihood of being detected, as attention is naturally biased toward certain regions of the visual field. Additionally, emotional valence can also influence detection and identification processes. Fairfield et al. (2022) found that positive stimuli enhance memory in older adults and improve target identification in young adults, particularly when images are briefly presented (100 ms), suggesting that emotional content influences early stages of processing.

The discrimination threshold, in turn, refers to the smallest difference necessary between two stimuli to perceive their distinction, that is, the minimum amount of change between stimuli that we can detect. When we talk about visual contrast, we highlight the difference in luminance between a stimulus and the background. According to Whittle (1986), for low contrasts, the discrimination threshold changes depending on the difference in luminance between the image and the background. However, for high contrasts, the threshold is proportional to the absolute luminance.

Additional studies corroborate the importance of contrast in visual perception. Einhäuser and König (2003) found that, in naturalistic images, luminance contrast does not contribute significantly to human visual attention. Recently, Rahimi-Nasrabadi et al. (2021) explored the impact of luminance contrast and environmental lighting on visual context learning and retrieval. Their findings revealed that increasing luminance contrast enhances the efficiency of contextual cueing, facilitating more effective visual searches. Additionally, they observed that context retrieval performed optimally under intermediate lighting conditions, whereas it declined under bright daylight due to the reduction of the contrast of luminance.

Stimulus detection in natural environments is a complex task that involves several contextual and dynamic factors. Much of the current literature on stimulus detection is based on tasks that use static stimuli presented against neutral backgrounds (Karas and McKendrick, 2015; Kumcu et al., 2015; Meinhardt et al., 2006). However, such conditions do not reflect the reality of the dynamic environments in which we live, where the visual context is constantly changing. This was demonstrated by Marks and Goard (2021), who studied brain activity in response to fixed/artificial and dynamic/naturalistic stimuli. They concluded that brain responses to fixed stimuli, such as gratings, remain stable over time, whereas responses to naturalistic stimuli continuously evolve in a dynamic manner. The use of naturalistic stimuli in neuroscience has been widely discussed due to their higher ecological validity and their potential to explore cognitive processes in real-world conditions (Sonkusare et al., 2019). While these stimuli provide a more realistic framework for understanding perception, they also have problems related to variable control and the need for more complex analytical methods. For example, Kumcu et al. (2015) investigated how image noise, as well as background luminance impact contrast detection thresholds. Their results indicate that noise affects contrast perception, modifying detection thresholds depending on the surrounding visual conditions. Although their study was conducted under less naturalistic conditions, its findings suggest that in real-world settings, where visual noise and background luminance are constantly shifting, contrast detection may become less predictable and more dependent on contextual factors. However, in everyday life, contrast perception occurs in dynamic environments, and using these natural stimuli is essential to understand how we detect contrast in the real world.

While many studies focus on the role of color in visual perception, research addressing the effects of luminance remains less explored. Bortolotti et al. (2022) conducted a review that compiled research on how color influences perception, also mentioning studies on luminance. One example they highlight is that natural light in a room can generate psychophysiological benefits, effects that are not observed in the absence of natural light. However, in their conclusion, they note that there is still insufficient research to fully describe how luminance affects cognitive performance. This highlights the need for further studies to better understand the role of luminance in perceptual processes. In this sense, the present study contributes to this discussion by investigating how different contrast levels—directly related to luminance—affect stimulus detection in a dynamic environment. By exploring this aspect, it helps expand the understanding of how luminance variations influence perceptual processes.

This study had as its main goal to tell how luminance contrast of the target stimulus against the dynamic background affects its detection threshold. Additionally, the timing and position of the event were analyzed to assess their impact on the probability of detection. Furthermore, this study intended to identify the contrast threshold for object perception. Since detecting an event does not mean actually perceiving it, an additional task of shape discrimination was implemented to identify the contrast threshold for perceptual formation. Perceiving shapes follows a hierarchical process, where the brain first detects edges and contrasts, then groups these elements into coherent shapes, and finally recognizes and categorizes objects (Loffler, 2008). In the early stages, contrast plays a key role in identifying basic visual features. As processing advances, shapes are organized, allowing for a more structured perception. Avidan et al. (2002) studied the reliance on contrast in the different stages of visual processing and demonstrated that early stages of visual processing are more contrast dependent whereas later stages become less influenced by contrast. Both studies conclude that early stages of visual processing are more dependent on contrast. Therefore, we hypothesized that a higher contrast would be needed for object discrimination than for event detection.

## Materials and methods

### Participants

There were 21 individuals (five male and 16 female) who took part in this study, with ages between 18 and 35. All participants were university students who were recruited through university announcements. They volunteered and received extra course credits as compensation for participation. All participants had normal or corrected-to-normal vision. All 21 participants signed an informed consent form. The experimental procedure was approved by the Ethics Committee of the University of the Azores.

### Stimuli

The background stimulus used consisted of a video acquired from the Pexels image database^1^, known for providing royalty-free videos. The chosen video was accessed through the search “A man walking under a bridge” and provided a natural dynamic environment for the presentation of the stimuli (images provided in Supplementary material). During the presentation of the background stimulus, visual objects were briefly presented (See Supplementary Figures 1, 2). These visual stimuli consisted of two distinct images: either a round bush or a rectangular one. The choice of natural stimuli, such as bushes, was to keep the stimuli naturalistic and integrated within the theoretical framework of natural scene perception. Additionally, bushes were selected as neutral stimuli, minimizing the potential for emotional biases that could influence detection and perception.

Each stimulus was inserted into the video either early on or later (at 360 ms for early stimuli and 3.80 s for late stimuli), varying its position and luminance contrast. The manipulation of the timing of the stimuli was meant to prevent predictability of stimuli, which might have increased detection and discriminability.

The bushes were manipulated to achieve different levels of contrast in relation to the luminance of the local background. The type of contrast calculation used was based on differences between absolute luminance levels, not color, due to color variations throughout the scenes. The selected contrast levels (20%, 40%, 60%, and 80%) were chosen to cover a wide range of perceptual difficulty, ensuring that the stimuli did not have excessively low contrasts, which could make detection extremely difficult, nor excessively high contrasts, which could result in immediate visibility. This range was carefully selected to provide a balanced variation, allowing for a meaningful analysis of detection and discrimination processes. The luminance of the exact visual areas where the stimuli appeared (background luminance) were measured before and after the insertion of the stimuli to ensure accurate contrast assessment. This process was repeated 20 times, and the average of the measurements was calculated for each contrast level. The calculation of the contrast percentage was based on the following formula:


Contrast=(Lo-Lb)/Lb100*

where Lo was the luminance of the object and Lb was the luminance of the background.

An example of the stimuli can be observed in Table 1.

**TABLE 1:** The round and rectangle-shaped stimuli at the several contrast levels.

Shape	Contrast at 20%	Contrast at 40%	Contrast at 60%	Contrast at 80%
Round	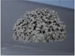	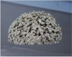	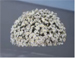	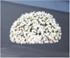
Rectangle	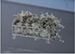	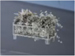	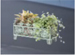	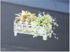

The stimuli were positioned in four different locations throughout the videos: in the lower left, the lower right, the upper left and the upper right corner of the video. These locations were selected to provide an even distribution of the stimuli and to spread visual attention through the entire scene, avoiding learning and expectation effects. Moreover, these arrangements allow for the exploration of asymmetries in visual processing. Previous research indicates that there are differences in attentional allocation across different areas of the visual field (Iyilikci et al., 2010; Nuthmann and Matthias, 2014; Hartmann et al., 2019; Chiffi et al., 2021). The stimulus appeared only during one frame of the video, which was played at 60 frames per second (fps) to ensure that the stimulus is close to the detection threshold.

The participant sat at 80 cm distance from the screen. The full visual scene subtended 61.93° of field of view (FOV) horizontally by 39.22° FOV. The round bush stimuli were 3.58° by 2.86° FOV, while the square bush stimuli were 4.30° by 2.15° FOV. The top stimuli had their center of mass at ∼16.36° FOV up and the bottom stimuli had their center of mass at ∼16.36° FOV down from the center of vision. The left stimuli were at 38° FOV to the left, while right stimuli were at 38° FOV to the right.

### Experimental design

This study employed a 2 × 4 × 2 × 4 factorial experimental design: the independent variables included two stimulus onset times, four stimulus-background contrast levels (20%, 40%, 60%, 80%), two shapes (square or round), and four stimulus positions. All stimuli were repeated three times. Participants were tasked with reporting the stimulus shape (rectangle or round) after each presentation of each trial, if a stimulus was detected.

### Materials

The luminance measurements and the experimental sessions took place in a laboratory room, ensuring absolute silence and the absence of external light, with walls covered in absorbing dark materials to remove light scattering and sound. Luminance measurements were performed with a photometer (Digital Lux Meter Dr.meter LX1330). The experimental tests were conducted using the *Psychopy* software, allowing the controlled and randomized presentation of visual stimuli to the participants. The videos with the stimuli were displayed on a 43-inch monitor, configured with the same brightness settings in all experimental sessions. The average background luminance of the presented scenes was 56 cd/m^2^ (SD = 3 cd/m^2^). To further prevent any participant distraction, white noise was presented during the totality of the experiment. Marshall Monitor M-ACCS-00152 headphones were used for the presentation of the white noise sound.

### Experimental procedure

The experiment consisted of 192 trials, with each video lasting 5 s, resulting in a total duration of approximately 15 min of continuous task execution without breaks. Of these, 50% (96 trials) contained a stimulus, while the remaining 50% did not contain any stimulus. Although the absence of pauses could introduce some fatigue, the relatively short duration of each trial helped mitigate its impact, allowing participants to sustain attention throughout the task. Each repetition was generated randomly, by manipulating the position, contrast, and shape of the visual stimulus. The stimulus exposure time was one frame (16.7 ms). Participants were instructed to detect the visual stimuli and respond using a standard computer keyboard. If a visual stimulus was identified as round, participants were asked to press the left Shift key; if it was identified as rectangular, they were asked to press the Enter key. In the absence of detection, participants were instructed to take no action.

### Data analysis

There were two dependent variables, each analyzed in a separate subsection of the section Results: Detection Thresholds and Shape Discrimination Thresholds. There were three independent variables: Time, Position and Contrast.

To obtain the detection and discrimination thresholds, cumulative Gaussian curves were fitted according to the bootstrap method by Foster and Bischof (1997) with 1,000 iterations. This is a commonly used method, which also provides an estimate for the perceptual thresholds and curve spread, as well as a statistical tool to run differential tests on two curves. The source code for this statistical test can be found in https://webdocs.cs.ualberta.ca/∼wfb/bootstrap.html.

A Kolmogorov-Smirnov test was run to assess if the data were normally distributed. Mixed Models ANOVAs were run to observe the effects of the independent variables on the dependent variables. *Post-hoc* Tukey tests were run where applicable.

## Results

### Detection thresholds

To analyze the detection rates, we quantified the number of responses obtained whenever a stimulus was present. Responses obtained in the trials when stimulus was absent were false alarms. False alarms occurred in 15% of the trials. Correct stimulus detection varied along the varying levels of stimulus contrast, and also with stimulus timing and position. Accounting for the distribution of false alarms and correct detections, we calculated the sensitivity index (d’) according to the following formula:


$$d^{\prime }=Z\,\left(H\right)-Z\,\left(F\right),$$

where *H* is the Hit rate, given by probability of saying the stimulus was present when stimulus was present, and *F* is the False alarm rate, or the probability of reporting a stimulus when no stimulus was present. *Z* indicates the reporting of the normalized values of each probability distribution function.

We also calculated the response bias (criterion C) for each contrast level and for early and late occurring stimuli (Table 2). This calculation was done as follows:


$$C=-\frac{1}{2}\,\left[Z\,\left(H\right)+Z\,\left(F\right)\right].$$

**TABLE 2:** Sensitivity index (d’) and response bias (Criterion C) according to stimulus contrast (20%–80%) and timing of presentation (Early or Late).

Stimulus contrast	d’ (early)	Bias (early)	d’ (late)	Bias (late)
20%	1.162	0.455	1.342	0.365
40%	1.449	0.312	1.914	0.079
60%	1.914	0.079	2.347	−0.137
80%	2.073	0.000	2.512	−0.220

The d’ index normalizes detection rates and accounts for false positives in the trials where stimuli were absent. Discriminability was always above 1, revealing above chance detections when stimuli were present. By increasing the contrast of the stimulus, sensitivity increased, albeit never reaching a ceiling performance. Response biases varied across conditions. It can be observed that late stimuli with high contrast had lower criteria, with values below 0, signifying a shift toward a more liberal bias.

The d’ values were always above 1, even at the baseline contrast of 20%, meaning that there was some sensitivity even at those levels. The sensitivity increased for all stimuli with increasing contrast, never reaching values that would denote a ceiling effect. Conversely, the bias values (*C*) were predominantly above 0, revealing a conservative bias, or a general tendency to not detect the stimuli. Interestingly, for the late events at the contrasts of 60% and 80%, the bias values dipped below 0 (−0.137 and −0.22, respectively), indicating a more liberal response criterion. This shift may, at least partly, explain the increase in responses for late, high contrast stimuli.

**FIGURE 1:**
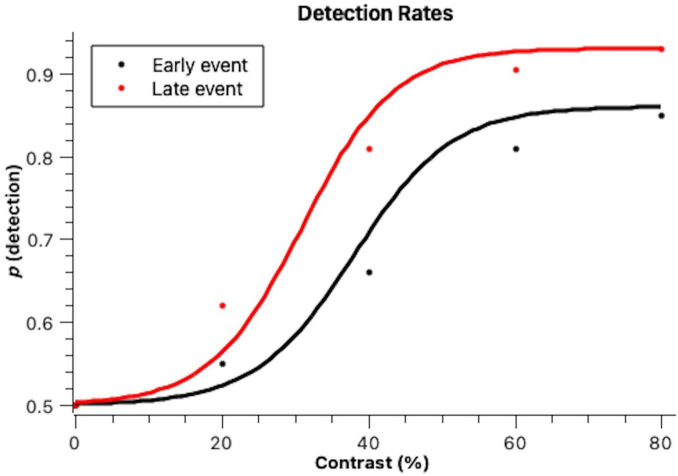
Proportions of detected stimuli when they were presented early on in the scene (Early event) and later on in the scene (Late event) across different figure-background contrast levels. Lines denote cumulative Gaussian fits to the data, according to the bootstrap method with 1,000 iterations by Foster and Bischof (1997). This is a well-demonstrated method for fitting psychophysical functions and to determine psychophysical parameters.

Indeed, as can be seen in Figure 1, the higher the contrast the higher the stimulus detection and late events were generally more detected. Data were fit to psychophysical functions according to the bootstrap method by Foster and Bischof (1997). The curves obtained had a good fit to the data (early: *r*^2^ = 0.994, *p* = 0.012; late: *r*^2^ = 0.995, *p* = 0.011). The two psychophysical functions have different slopes, meaning that stimulus timing affected discriminability: early events were less discriminable than late events. The bootstrap thresholds were statistically different at the 0.05 level, set at 35% contrast for late stimuli and at 43% for early stimuli (average = 39%). The late events were more detected overall and reached higher maximum detection levels at 80% contrast, with 94% of the events being detected. The early events were less detected at 80% contrast were only detected in 88% of the trials.

A Kolmogorov-Smirnov test determined that the detection rates were normally distributed. A Mixed Models ANOVA with stimulus detection as the dependent variable revealed a significant effect of contrast [*F*_(3)_ = 15.88, *p* < 0.001]. In *post-hoc* pairwise comparisons (Tukey), it was observed that contrast levels differed between each other regarding detection rates. Stimulus timing also affected its detection, with earlier events being less detected than later events [*F*_(1)_ = 6.71, *p* = 0.010].

**FIGURE 2:**
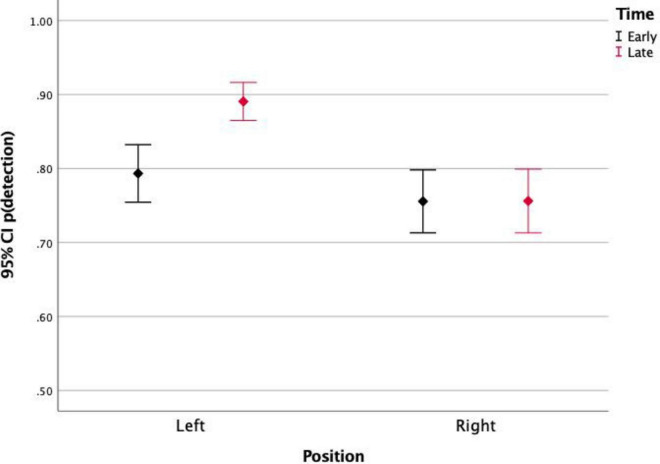
Proportion of detections of stimuli presented on the left and on the right side of the screen, for early and late events. Left events were more detected, especially those presented later.

**FIGURE 3:**
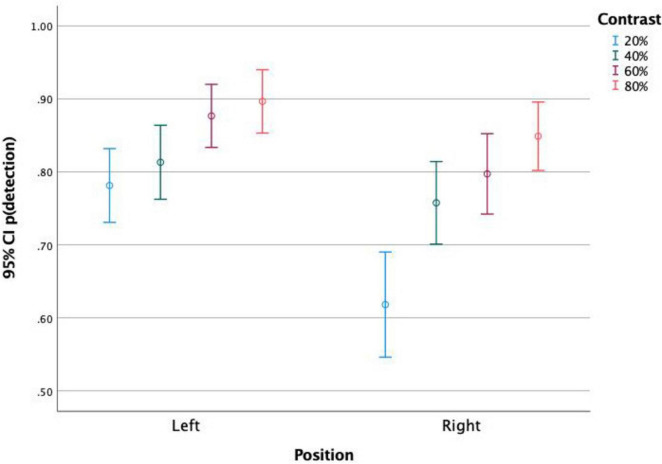
Proportion of detected stimuli presented on the left and on the right side of the screen, for each stimulus contrast level. It is observed that stimuli to the left were more detected than stimuli to the right at similar contrasts. The higher the contrast, the higher the detection level. Both stimulus position and contrast affected detection rates.

**FIGURE 4:**
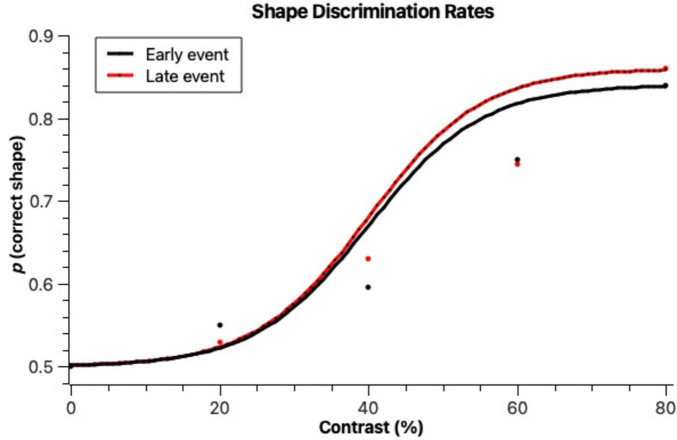
Proportions of correctly identified stimulus shapes when they were presented early on in the scene (Early event) and later on in the scene (Late event) across different figure-background contrast levels. Lines denote cumulative Gaussian fits to the data, according to the bootstrap method by Foster and Bischof (1997). It is observed that contrast levels affect shape discrimination accuracy, but the timing of evens (Early vs. Late event) does not.

Finally, the stimulus position significantly affected the detection rates [*F*_(1)_ = 21.09, *p* < 0.001]. This effect was not significant for the differences between detections of stimuli in top and bottom positions, but it was significant for left and right positions. Figures 3, 4 show the detection rates plotted separately for left and right stimuli. It is observed that stimuli displayed in the left corners of the scene were more detected than stimuli displayed in the right corners. When analyzing together the effects of stimulus position and stimulus timing (Figure 2), it is observed that the timing only affected the detection of stimuli presented in the left portion of the screen. For those stimuli, detection was significantly higher for late events (89%) than for early events (79.6%).

When analyzing together the effects of stimulus position and stimulus contrast (Figure 3), it is observed that both variables affected the rates of detection in all parameter levels, with a constant benefit of the stimuli to the left hemifield and of higher contrasts.

### Shape discrimination thresholds

Participants identified mostly “round” shapes when the stimuli were round (81.7% of trials) and “rectangle” shapes when stimuli were rectangular (68.1% of trials). There was, however, an asymmetry where there was a slightly larger tendency to answer “round” than “rectangle” (See Table 3). A Chi-square test revealed that the differences in proportions of “round” and “rectangle” answers did not reach statistical significance (|^2^_(1)_ = 2.71, *p* >0.05).

**TABLE 3:** Percentage of no-answer, round, and rectangle answers when round and square stimuli were present.

Answer		No answer	“Round”	“Rectangle”
Stimulus shape	Round	7.9%	81.7%	10.4%
	Rectangle	8.1%	23.7%	68.1%
Average		8.0%	52.7%	39.3%

To analyze shape discrimination thresholds, we quantified the number of correctly identified shape responses (rectangle or round) per trial where a stimulus was present. As can be seen in Figure 4, the higher the contrast, the higher the shape discrimination accuracy. Both early and late events had similar shape discrimination levels. At 20% contrast, discriminations were still at a rate close to random, with only 53% and 55% correct answers in the early and late event trials, respectively. The highest discrimination accuracy was obtained with late events at 80% contrast (86% correct). Early event trials only obtained a maximum of 84% correct trials at 80% contrast. The psychophysical functions observed in Figure 4 obtained a good fit (early: *r*^2^ = 0.987, *p* = 0.0344; late: *r*^2^ = 0.993, *p* = 0.0184). They do not differ between each other in terms of bootstrap threshold (*p* ≤ 0.05 level), but the slopes are significantly steeper for late events, revealing perhaps an increased sensitivity and less noisy judgements in those trials. The thresholds for correct shape identification were, according to the psychophysical functions (bootstrap method by Foster and Bischof (1997), 40% for the late events and 41.5% for early events. These thresholds were significantly different from the thresholds obtained for stimulus detection (*p* ≤ 0.01).

A Kolmogorov-Smirnov test determined that the correct shape identification rates were normally distributed. A Mixed Models ANOVA having correct shape identification as dependent variable and contrast, timing and position as independent variables was run. Stimulus contrast had a significant effect on shape discrimination accuracy [*F*_(3)_ = 7.15, *p* < 0.001]. Responses on 20% trials did not differ significantly from responses on 40% trials, but all other pairwise comparisons (Tukey test) across different contrasts revealed significant differences. Stimulus timing (early or late) did not yield significant differences in response accuracy [*F*_(1)_ = 0.05, *p* = 0.82, n.s.], as opposed to what was observed with stimulus detection.

Stimulus position had a significant effect on shape identification [*F*_(1)_ = 26.04, *p* < 0.001].

**FIGURE 5:**
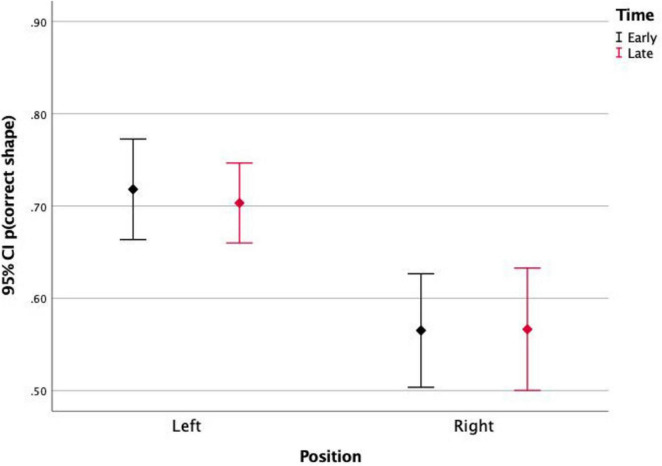
Proportion of correct shape discrimination and 95% Confidence Intervals of stimuli presented on the left and on the right side of the screen, for early and late events. It is observed that the position of the stimulus (left vs. right) affects its shape discrimination accuracy, but the timing (Early vs. Late) does not.

**FIGURE 6:**
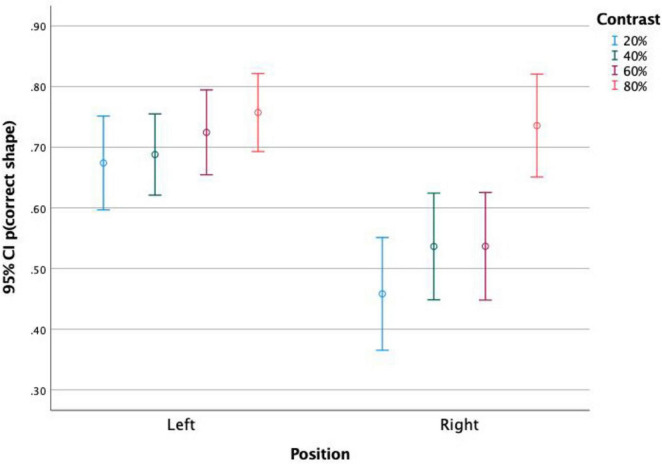
Proportion of correct shape discrimination and 95% Confidence Intervals. Discrimination of stimuli presented on the left and on the right side of the screen, for each stimulus contrast level. It is observed that both the positioning of the stimulus and its contrast affect the rate of shape discrimination accuracy.

This effect was not significant between stimuli in top and bottom positions, but it was significant between left and right positions. Figures 5, 6 show the correct shape discrimination rates plotted separately for left and right stimuli. It is observed that stimuli displayed in the left side of the scene were more correctly perceived (average 71.3%) than stimuli displayed in the right side (average 56.5%). While stimulus timing did not change the shape perception (Figure 5), stimulus contrast did (Figure 6).

## Discussion

The main goal of this study was to quantify the impact of contrast levels on the detection and shape perception of brief events in natural dynamic scenes.

Stimulus detection rates were found to be affected by contrast level, which confirmed our main hypothesis. Indeed, this result is backed by previous studies showing that the detection of visual stimuli depends on their contrast (e.g., Pelli and Bex, 2013; Park et al., 2017).

The threshold for event detection was found to be on average 39% contrast, but several factors were found to affect it. Events presented earlier in the scene had a higher threshold, while events presented later in the scene had a lower contrast threshold. This effect is explained, at least in part, by a change in participant bias toward a greater tendency to detect stimuli, as shown in Table 2.

This effect might also reveal some higher-order, non-stimulus-driven effects, such as attention, memory, or expectation, since the stimulus remained equally salient in both conditions. Hu and Mohsenzadeh (2024) showed that high-level information is resolved in later time. This means that only low-level information is processed early on, and complex stimuli such as a 1 frame image in a complex video may take longer to process which is why the detection early on could be worse.

It was also found that stimuli presented on the left side of the screen were more detected than stimuli presented on the right side. This difference was more accentuated in late occurring events. This effect of stimulus position also points towards attention-driven factors. In fact, in a linear regression model accounting for all variables, more detection was predicted by left-right stimulus positioning than by contrast variations. The asymmetry in detection rates can indeed be attributed to differences in attentional processing and sensory perception. Research indicates that visual processing asymmetries, particularly in change detection tasks, reveal a left visual field advantage, suggesting that attentional mechanisms play a critical role in how changes are perceived across different visual fields (Iyilikci et al., 2010; Nuthmann and Matthias, 2014; Hartmann et al., 2019; Chiffi et al., 2021).

Regarding shape discrimination levels, the timing of events did not show significant effects. Once again, events occurring on the left hemifield were more accurately discriminated than events on the right. Regarding the bootstrap thresholds, it was observed that the threshold required for the correct identification of shapes is 40.8%, higher than the level necessary for stimulus detection. In fact, visual detection and shape perception involve distinct underlying neuronal mechanisms, with perception requiring more complex cognitive processes and a slower processing pathway (Loffler, 2008; Koivisto et al., 2017). Marr (1982) describes that processing occurs in three distinct levels. At the first level, the brain detects the most basic visual features, such as edges, contours, and contrasts, which are essential for the initial detection of stimuli. This process is quick and straightforward, allowing the identification of a stimulus. It is possible that lower contrasts may be sufficient at this initial level to detect edges and simple stimuli. However, shape discrimination, which involves identifying and differentiating complex shapes, requires more elaborate processing. At the second level, the brain groups these basic features to form contours and more complex patterns, where higher contrasts may be necessary to support this process. Finally, at the third level, these representations are analyzed and interpreted to recognize specific shapes. This level may depend on the quality and accuracy of the information processed in the earlier levels, suggesting that insufficient contrast could limit the clarity of transmitted information, negatively impacting shape discrimination capabilities (Iyilikci et al., 2010; Nuthmann and Matthias, 2014; Hartmann et al., 2019; Chiffi et al., 2021).

In sum, this study identified some parameters affecting detection and discrimination of brief visual stimuli presented over a complex and dynamic, naturalistic scene. Contrast levels above 40% should be detectable in more than 50% of instances. Other factors were found to interact with contrast, namely the timing of the event and its position in space. Later stimuli have lower thresholds than earlier stimuli, and stimuli on the left are more detected and better discriminated than stimuli on the right.

### Limitations and future directions

The findings of this study were based on a specific sample, which may limit their generalizability. Future research should explore age-related differences, cultural variations in visual perception, or clinical populations, such as individuals with attentional deficits.

Additionally, this study used a single natural dynamic scene, which has specific visual properties that influence stimuli detection and discrimination. Future studies could examine a wider range of visual environments (e.g., closed spaces, different types of landscapes, or urban settings) to determine whether the observed effects persist across various contexts.

Another limitation is that only one type of stimulus was used. In future research, variations in shape or size could be introduced to investigate whether the observed effects generalize to different types of visual stimuli.

Finally, future research could explore contrast with eye-tracking techniques to examine how fixation patterns, saccades, and pupil responses are influenced by different contrast levels. These studies could investigate whether high-contrast areas attract attention more efficiently, how contrast impacts visual search in dynamic environments, and whether cognitive load affects the ability to detect low-contrast stimuli.

## Conclusion

The findings of this study provide insights into how contrast influences visual perception and stimulus detection, with implications that extend beyond controlled environments. These results can be applied to real-world contexts such as traffic safety, public signs, and architectural design, ensuring that critical visual information is easily perceivable. For example, in the design of visual displays, such as information boards at airports or train stations, digital panels, and emergency alert systems, optimizing contrast can enhance detection and speed up the identification of information, ensuring that users efficiently perceive essential visual stimuli. The findings of this study suggest that adequate contrast levels are crucial for ensuring that critical information is quickly perceived, reducing the time needed to interpret visual messages and make decisions in situations where response time is critical. Furthermore, in safety systems, these insights can inform the development of high-visibility warning signs, workplace hazard indicators, and emergency evacuation routes, ensuring that essential visual cues remain detectable under varying lighting conditions. Determining the optimal contrast levels for natural situations is essential to ensuring both safety and visual comfort, balancing visibility in critical environments while preventing visual fatigue and overstimulation in everyday contexts.

## Data Availability

The raw data supporting the conclusions of this article will be made available by the authors, without undue reservation.

## Footnotes

1
https://www.pexels.com/video/a-man-walking-under-a-bridge-5362053/
