# Supplementary figures and images for: Contrast affects stimulus detection in natural scenes
Source: Front Hum Neurosci. 2025 May 15;19:1553504. doi: 10.3389/fnhum.2025.1553504 (PMC12120249; doi:10.3389/fnhum.2025.1553504)

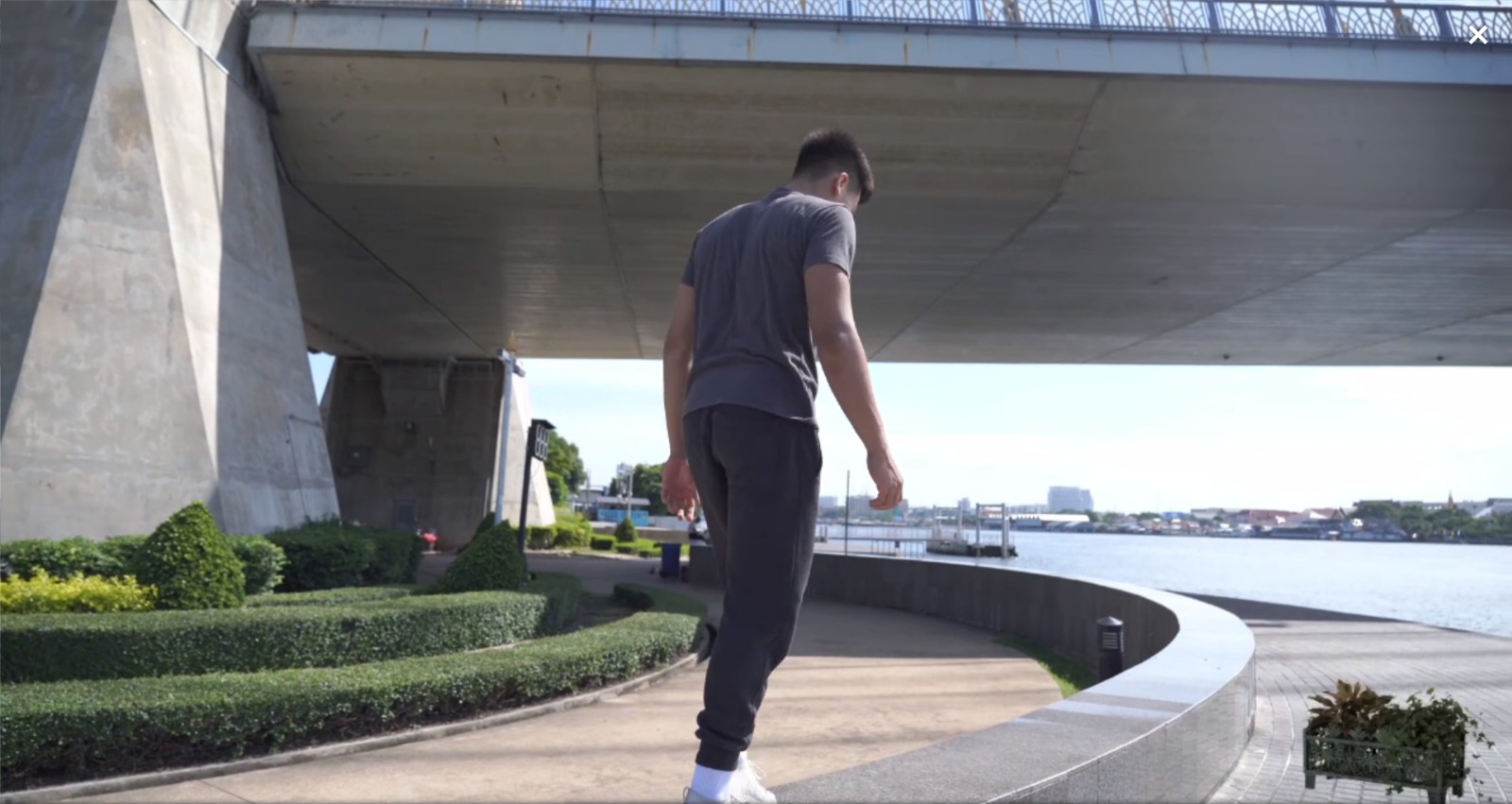

Supplement: Supplementary file 1 [file Image_1.jpeg]

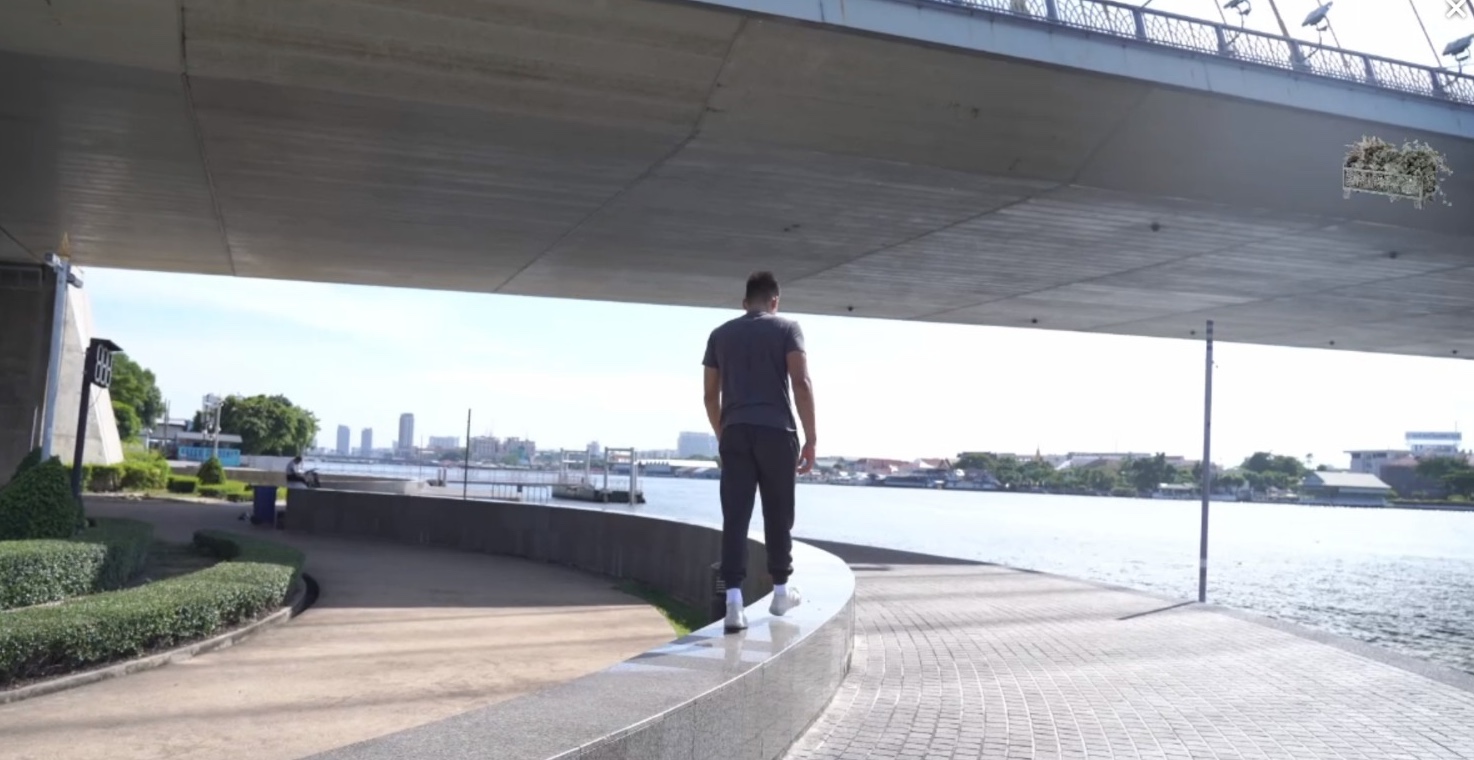

Supplement: Supplementary file 2 [file Image_2.jpeg]
